# A New Threat to Honey Bees, the Parasitic Phorid Fly *Apocephalus borealis*

**DOI:** 10.1371/journal.pone.0029639

**Published:** 2012-01-03

**Authors:** Andrew Core, Charles Runckel, Jonathan Ivers, Christopher Quock, Travis Siapno, Seraphina DeNault, Brian Brown, Joseph DeRisi, Christopher D. Smith, John Hafernik

**Affiliations:** 1 Department of Biology, San Francisco State University, San Francisco, California, United States of America; 2 Department of Biochemistry and Biophysics, University of California, San Francisco, San Francisco, California, United States of America; 3 Entomology Section, Natural History Museum of Los Angeles County, Los Angeles, California, United States of America; Royal Holloway University of London, United Kingdom

## Abstract

Honey bee colonies are subject to numerous pathogens and parasites. Interaction among multiple pathogens and parasites is the proposed cause for Colony Collapse Disorder (CCD), a syndrome characterized by worker bees abandoning their hive. Here we provide the first documentation that the phorid fly *Apocephalus borealis*, previously known to parasitize bumble bees, also infects and eventually kills honey bees and may pose an emerging threat to North American apiculture. Parasitized honey bees show hive abandonment behavior, leaving their hives at night and dying shortly thereafter. On average, seven days later up to 13 phorid larvae emerge from each dead bee and pupate away from the bee. Using DNA barcoding, we confirmed that phorids that emerged from honey bees and bumble bees were the same species. Microarray analyses of honey bees from infected hives revealed that these bees are often infected with deformed wing virus and *Nosema ceranae*. Larvae and adult phorids also tested positive for these pathogens, implicating the fly as a potential vector or reservoir of these honey bee pathogens. Phorid parasitism may affect hive viability since 77% of sites sampled in the San Francisco Bay Area were infected by the fly and microarray analyses detected phorids in commercial hives in South Dakota and California's Central Valley. Understanding details of phorid infection may shed light on similar hive abandonment behaviors seen in CCD.

## Introduction

The honey bee *Apis mellifera* has experienced recent unexplained die-offs around the world [1]. In the United States, Colony Collapse Disorder (CCD), a syndrome characterized by loss of hives and the behavior of hive abandonment, threatens honey bee colonies and has received considerable scientific and media attention. While the United States is the only country for which CCD *sensu stricto* has been documented, there also has been an increase in unexplained colony losses for some regions of Europe and other parts of the world [1]–[4]. At the same time, some regions of Europe and Asia have reported only normal colony losses. Although catastrophic losses of honey bee colonies have occurred in the past, the magnitude and speed of recent hive losses appear unprecedented [1]. So far, the main causal suspects have been parasitic mites, fungal parasites, viral diseases and interactions amongst them [1]–[5]. While viral and microsporidian infections have been linked to increased mortality and declining health in honey bee colonies [5], [6], studies have not directly addressed behavioral changes involved in abandonment of hives.

Honey bees suffer from numerous parasites and pathogens including viruses, bacteria, parasitic fungi and ectoparasitic mites [7]. Infections from agents within any of these pathogen and parasite groups can be fatal to honey bees, but the parasitic *Varroa destructor* mite appears to be the most harmful to colonies overall. *Varroa destructor* is widespread in honey bee hives, affecting every life stage of honey bees from larva to adult [8]. Probably because of this, beekeepers in the United States rank parasites as a bigger threat to their honey bee colonies than CCD [1]. Controlling for parasitic mites is time consuming and costly with damage control estimated in the billions of dollars worldwide [9]. Further, *V. destructor* has been implicated as a vector of many pathogens that can compromise colony health [10]–[12]. Understanding parasitic infections in honey bees is crucial in predicting the long-term health of honey bee hives.

Here we report that *Apocephalus borealis*, a phorid fly native to North America, previously known to parasitize bumble bees and paper wasps [13], [14], also attacks the non-native honey bee. The genus *Apocephalus* is best known for the “decapitating flies” that parasitize a variety of ant species [15]. *Apocephalus borealis* belongs to the subgenus *Mesophora*, which is a group that contains species that attack hosts other than ants. Although the hosts of most species in the *Mesophora* group are unknown, previously discovered hosts include a variety of arthropods including bees, wasps, beetles and spiders, but not honey bees [14].

In this paper, we show that *A. borealis* has a profound effect on parasitized honey bees, leading them to abandon their hives at night. We use an Arthropod Pathogen Microarray (APM) [16] to detect pathogens that have been implicated in CCD that are associated with adult flies and larvae and to detect the presence of phorids in commercial hives in South Dakota and California's Central Valley. Understanding causes of the hive abandonment behavior we document could explain symptoms associated with CCD. Further, knowledge of this parasite could help prevent its spread into regions of the world where naïve hosts may be easily susceptible to attack.

## Results

**Figure 1:**
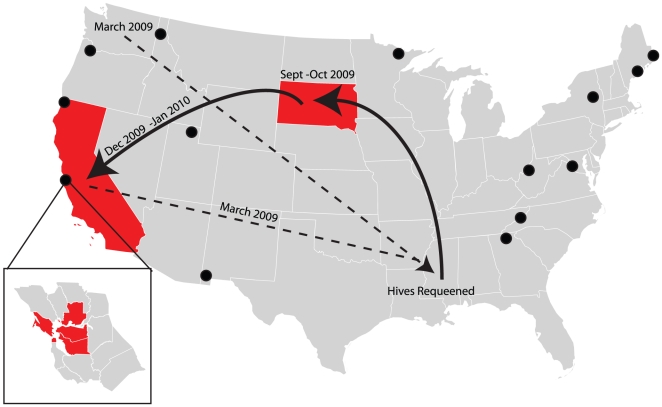
Distribution of phorid-infected honey bees sampled in this study (red). Inset shows the San Francisco Bay Area counties where we found phorid-parasitized honey bees. The routes of commercial hives tested are indicated (arrows), where dotted lines represent states the hives crossed before viral microarray testing and solid lines represent the route of hives during the period of microarray testing. Sites where *A. borealis* was previously known [7] are indicated by black dots.

We found widespread parasitism by *A. borealis* amongst 7,417 honey bees and 195 bumble bees (177 *Bombus vosnesenskii*, 18 *Bombus melanopygus*) sampled from San Francisco Bay Area localities (Figure 1 and Table S1). In all, 77% of our sample sites (24 of 31) yielded honey bees parasitized by *A. borealis*. We reared phorids from 26 *B. vosnesenskii* workers, one *B. vosnesenskii* queen and one *B. melanopygus* worker.

Using DNA barcoding, we confirmed that the phorids that emerged from *Apis* and *Bombus* had no more than 0.2% (1 bp) divergence among samples (Figures S1, S2). The slight variation we found was among those phorids reared from honey bees, not between flies reared from honey bees and those reared from bumble bees. We further confirmed the identity of the phorids using morphological criteria and sequencing of 18S rRNA genes used on the APM. In addition, our lab infections of honey bees (see below) used phorids that had emerged from both honeybees and bumblebees. Flies from both hosts responded in the same way to the presence of honey bee workers. Taken together these data confirm that the phorids that attack honey bees are the same species as those attacking bumble bees.

Foraging *B. vosnesenskii* showed a higher rate of phorid parasitism than *A. mellifera* foragers (Table S3). Although our individual sample sizes for bumble bees are small due to their relative rarity in summer 2010, we observed parasitism rates as high as 80% (8/10) in one sample of foraging bumble bees from September.

**Figure 2:**
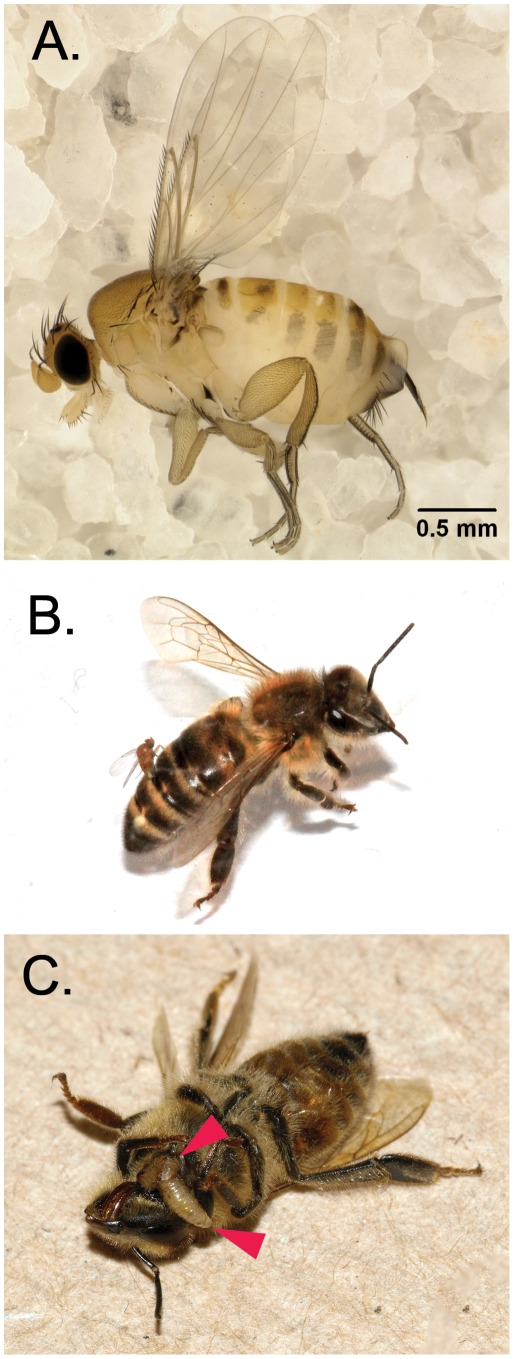
Images of *Apocephalus borealis* and honey bees. (A) Adult female *A. borealis*. (B) Female *A. borealis* ovipositing into the abdomen of a worker honey bee. (C) Two final instar larvae of *A. borealis* exiting a honey bee worker at the junction of the head and thorax (red arrows).

In laboratory infections, female flies attacked honey bees soon after they were placed in an arena with them. Female flies pursued a bee, landed on its abdomen and inserted their ovipositors into it for two to four seconds (Figure 2A, 2B). We observed the same behavior towards honey bees from phorids reared from bumble bees or from honey bees. This interaction is similar to that of other species of phorids that parasitize ants [17] and bees [18]. Mature phorid larvae emerged from the junction between a bee's head and thorax (Figure 2C), on average, seven days after collection (n = 636, Range = 1–14, SD = 1.68) (Figure S3A) and moved away from the bee to pupate. All larvae that emerged from worker bees successfully pupated under laboratory conditions (see methods). Production rates from field-collected bees ranged from one to 13 mature larvae per infected bee (n = 961, Mean = 4.8, SD = 2.4) (Figure. S3B), giving flies the potential to multiply rapidly. In the laboratory, we observed even higher maximal larval production with one bee producing 25 pupae. Adult flies emerged on average in 28 days (n = 94, Range = 22–36, SD = 1.9) after pupation (Figure S3C).

To investigate internal hive behavior and possible infections within a hive, we kept an observation hive in a laboratory near our primary study hive. Samples taken from the observation hive in June 2010 confirmed infection with *A. borealis*. Rates of infection varied between June 2010 and December 2010 (Mean = 25% Range = 12%–38%) peaking over the sample period in November at 38%. In September, the number of bees in the hive declined and we observed phorid pupae and empty pupal casings among dead bees at the bottom of the hive, indicating emergence of adult phorids within the hive and the potential for *A. borealis* to multiply *within* a hive and infect a queen.

Using an Arthropod Pathogen Microarray (APM) [16], we detected four phorid-positive samples which also shared 99.8% identity over a 432 nt fragment of the 18S rRNA gene (Figure S2) from bees in traveling commercial hives: two from South Dakota during September and October of 2009 and two near Bakersfield, California in January and February of 2010 (Figure 1) [16]. Notably, the APM also detected a higher rate of apparent phorid infection in samples from San Francisco State University on dates when larval emergence assays measured lower levels of parasitism. In this regard, array samples collected between April 23 and June 18, 2010 from various locations on campus (Table S2) detected phorids in 10 of 31 bees (32%) versus only 17 of 244 (7%) detected by our emergence assays (Fishers Exact Test p<0.0002). This difference suggests that the APM is the more sensitive tool to measure infection rates and that our emergence assay data provide a conservative estimate of the abundance of phorids.

We screened phorid adults, larvae and parasitized bees for honey bee pathogens with the APM [16], [19]. Phorid adults and phorid larvae tested positive for infection by *Nosema ceranae* (4/8 adults and 7/8 larvae) and deformed wing virus (DWV) (2/8 adults and 6/8 larvae) (Table S2). Bees from monitored hives and stranded bees sampled from a variety of locations were commonly infected with *N. ceranae* (26/36 bees), and DWV (16/36 bees). Presence of nucleic acid from these pathogens indicates that particles are present, not that they are replicating or are in an infectious form.

**Figure 3:**
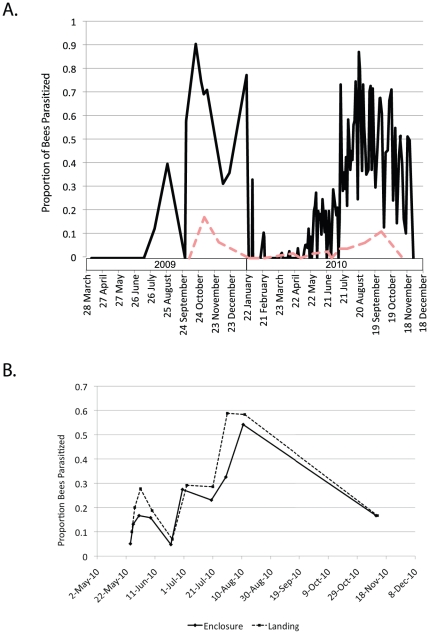
Rates of phorid parasitism in honey bees. (A) Rates of parasitism for bees sampled from April 2009 through November 2010. Black solid line shows rates in stranded bees from under lights on the San Francisco State University campus, while the pink dashed line shows rates in foraging bees. Stranded bees found under lights were sampled at irregular intervals during 2009 and sampled every two days in 2010. Foragers were sampled monthly from our main study hive. A rate of zero indicates that samples from that period contained no parasitized bees. We compared rates of parasitism in stranded and active foraging bees collected at San Francisco State University from October 2009 through January 2010 and from July 2010 to December 2010 (when parasitism rates peaked). 2009–2010 peak rates of parasitism in samples of stranded bees (Mean = 60%, n = 276) were significantly higher than peak rates of parasitism in active foragers from our main study hive (Mean = 6%, n = 164) (χ^2^ = 126.7, d.f. 1, p<0.0001). This pattern repeated in 2010 when peak rates of parasitism in samples of stranded bees (Mean = 50%, n = 860) were again significantly higher than rates of parasitism in active foragers (Mean = 4%, n = 422) (χ^2^ = 255.3, d.f. 1, p<0.0001). (B) Proportion of honey bees parasitized by phorids in samples from stranded bees collected from the Hensill Hall landing under lights (dotted line) and from samples of bees collected from overnight hive enclosures on adjacent nights (solid line). Parasitism rates of bees trapped in the enclosures closely track rates in stranded bees found under lights during the same period and the number of bees found under lights significantly declined when the enclosure was in place (Welch's t-test p<0.0001) indicating that stranded bees came from our main study hive and were parasitized prior to abandoning the hive.

While there are previous reports of night activity in honey bees [20], we are the first to link night activity to hive abandonment. We first found stranded worker honey bees beneath lights and within light fixtures on the campus of San Francisco State University (37°43′24.9″N×122°28′31.93″W) (Figure S4A–C) under a variety of weather conditions including cold rainy nights when virtually no other insects were seen around lights. Stranded bees showed symptoms such as disorientation (walking in circles) and loss of equilibrium (unable to stand on legs). Unlike most insects attracted to light, stranded bees remained mostly inactive the next day until they died. Honey bees that left their hives at night had a much higher rate of parasitism by *A. borealis* than bees foraging during the daytime (χ^2^ = 133, d.f. 1, p<0.0001) (Figure 3A). From October 2009 to January 2010 parasitism rates were as high as 91% in one sample of nocturnally active bees with a mean parasitism rate of 63% for that period (SD = 18.5, Range = 32%–91%, n = 266 bees) (Figure 3A). During the same period, foraging bees collected at the hive had a mean parasitism rate of only 6% (SD = 8.2, Range = 0%–17.4%, n = 162 bees) (Figure 3A). Phorid parasitism declined from February through spring 2010 before climbing in May and peaking again in autumn 2010 (Figure 3A and Figure S5). During this second recorded peak of parasitism (July 2010–November 2010), stranded bees again had a significantly higher rate of parasitism than foragers (χ^2^ = 255.3, d.f. 1, p<0.0001). Parasitism rates in stranded bees again peaked at nearly 90% (Mean = 50%, SD = 19, Range = 11%–88%, n = 860 bees) while foragers had a much lower rate of parasitism (Mean = 4%, Range = 0%–11%, n = 422 bees). These peaks in infection occurred just prior to or during the time of year when losses of honey bee colonies from CCD and other causes peak in the San Francisco Bay Area.

We periodically placed an enclosure over our primary study hive and assessed rates of parasitism of bees that left their hive at night (Figure S4D). Samples of bees trapped in the enclosure (n = 10 samples) ranged from 24–62 bees per night (Mean = 43.5, SD = 15.4). These samples closely tracked the rates of parasitism of stranded bees under nearby lights sampled the day after the enclosure was in place (Figure 3B). Moreover, the number of stranded bees under lights each night significantly declined when the enclosure was in place (Mean = 0.8, SD = 1.14, Range = 0–3, n = 8) compared to a mean of 15.7 (SD = 7.26, Range = 6–29, n = 157) stranded bees for non-enclosure nights (Welch's t-test, p<0.001). This indicates that stranded bees primarily came from our main study hive. The few bees we found stranded on nights when the enclosure was in place probably came from our nearby observation hive. These data confirm that nocturnally active bees were parasitized before leaving their hive and were drawn to the nearby light.

## Discussion

The behavior we observed in honey bees is similar to that reported for imported fire ants, *Solenopsis invicta* parasitized by the phorid, *Pseudacteon tricuspis* [21], and suggests that *A. borealis* is manipulating the behavior of its host bees. Such host manipulation has been proposed as an adaptive evolutionary strategy for a number of interactions between a variety of parasites and their hosts [22]. Recent work on gypsy moth larvae infected with nucleopolyhedrovirus identifies the genetic mechanism of host manipulation. The virus manipulates larval behavior inducing larvae to climb to the tops of trees where they die, liquefy and rain virus on the foliage below to infect new hosts [23]. This study provides a clear example of modifications to the expression of a key gene in a host and supports the extended phenotype theory proposed by Richard Dawkins [24], [25]. In the case at hand, perhaps *A. borealis* manipulates the behavior of honey bees by changing a bee's circadian rhythm, its sensitivity to light or other aspects of its physiology. In order to show that the changes in bee behavior that we document are adaptive for the fly, future studies will need to document that the change in behavior leads to an increase in the fitness of the parasite [22]. Alternatively, phorid infection may be one of several stressors resulting in aberrant nighttime activity (Figure S5). If true, sick bees may altruistically leave their hives to reduce risk to hive mates [26]. A similar response has been proposed for bumble bees parasitized by conopid flies [27] and ants infected by a fungal pathogen [28]. If this explanation is correct, bees might also leave their hive in response to infections such as those that we detected using the APM. Hive mates might also detect parasitized bees due to behavioral or physiological changes associated with parasitism and eject them from the hive. For example, Richard *et al.* [29] showed that bees intentionally infected with bacterial lipopolysaccarides expressed significantly different cuticular hydrocarbon profiles compared to healthy bees and that coating healthy bees with the hydrocarbon profile of infected bees aroused significant aggression towards those bees by hive mates. If parasitism by *A. borealis* alters a bee's chemical signature, this could provide a means for workers to detect phorid-infected hive mates.

Our data clearly show that phorid-parasitized bees demonstrate the unusual behavior of abandoning their hives at night. However, we can't exclude the possibility that some parasitized bees also abandon their hive during normal foraging times and die at some distance from the hive. Future experimental studies comparing the daily activity patterns of parasitized versus unparasitized workers are needed to test this possibility.

Until now, North American honey bees have appeared relatively free of parasitoid insects [30], [31]. In South and Central America, honey bees are attacked by numerous species of phorid flies, almost none of which occur in North America [32], [33]. Our study establishes *A. borealis* as a novel parasite of honey bees and documents hive abandonment behavior consistent with a symptom of CCD. This is a cause for concern because other species of phorid flies can dramatically affect social insect behavior and are used as biocontrol agents of introduced fire ants [21], [34]–[36]. So far, our main study hive has persisted despite losses to phorid parasitism and infection from a variety of pathogens. Seasonal variation seen in the rates of parasitism in our main study hive is consistent with other honey bee diseases [16], but the relationship, if any, is not fully understood. Seasonal variation could be associated with the life cycle of the fly in which rates of parasitism of honey bees fluctuate as *A. borealis* populations seasonally increase and decline. The fact that we did not find fly adults within hives may indicate that phorids do not survive in large numbers during the late winter when foraging bees are inactive. A detailed study of a larger sample of hives is needed to measure effects of various densities of phorid parasitism on hive health.

It is possible that *A. borealis* expanded its host range to include the non-native honey bee many years ago and has gone unnoticed because infected bees abandon their hive and flies occurred undetected in low densities. We believe it is more likely that the phenomenon we report represents a recent host shift and an emerging problem for honey bees. Honey bees are among the most studied insects in North America due to their importance to agriculture. The meticulous attention given to honey bees by humans suggests that phorids would have been detected sooner had the host shift occurred long ago, especially since detection of the parasite does not require sophisticated techniques. Observation of dead bees over as little time as five days should detect phorid presence. Furthermore, honey bees have inhabited areas adjacent to electric lights for at least a century, yet we know of no reports of large numbers of honey bees aggregating around lights until recently. This latter point suggests that, even if the flies were present in low numbers in honey bee colonies in the past, something has happened recently that has increased densities making phorids an emerging threat. To test for the presence of phorids in honey bees at earlier times, the APM could be used to analyze preserved honey bees from previous decades. Additional studies of the distribution and frequency of phorid parasitism of honey bees in North America are needed to assess the scope of this phenomenon and to detect if it is expanding to other areas or is already widespread. The easiest way to monitor nocturnal abandonment of hives is to place light traps nearby and then monitor trapped bees for emergence of phorid larvae. We hope that our study and methods will enable professional and amateur beekeepers to collect vital samples of bees that leave the hive at night, in order to determine if these bees are parasitized by phorids.

The host shift from bumble bees to honey bees has potentially major implications for the population dynamics of *A. borealis*. Bumble bees live in relatively small colonies that last only a single season with only queens overwintering. Honey bees, on the other hand, live in much larger colonies with tens of thousands of individuals living in hives that are warm even in winter. If these flies have or can gain the ability to reproduce within hives they could greatly increase their population size and levels of virulence. Moreover, hundreds and sometimes thousands of commercial honey bee colonies are often found in close proximity to one another in agricultural areas. Such high host density might lead to population explosions of the fly and major impacts on the hives they parasitize. Further, *A. borealis* is already widely distributed across North America [14] (Figure 1).

Although we did not sample hive bees such as nurses to determine if these workers are being parasitized within the nest, infection rates in foragers alone may still have a strong affect on overall hive health. Koury *et al.* [37] modeled colony population dynamics and predict that significant loss of foragers (beyond a certain threshold) could cause rapid population decline and colony collapse. Their model also predicts that significant loss of foragers leads to hive bees moving into the foraging population at younger ages than normal accelerating colony failure. While our emergence data indicated relatively low infection rates by the fly, our APM data suggest infection rates that are considerably higher. If parasitized bees are numerous or co-occur with other infections, a hive could reach a tipping point leading to its collapse. The detection of *A. borealis* in bees from South Dakota and Bakersfield, CA underlines the danger that could threaten honey bee colonies throughout North America. Movement of commercial hives could quickly spread phorid infection; especially given the number of states that commercial hives cross and are deployed in.

Detection of DWV and *N. ceranae* in adult *A. borealis* raises a number of questions. Do these pathogens have a negative influence on the vitality of the flies or affect their behavior? In this regard, microsporidian infections reduce viability in some insect parasitioids [38] but not in phorid parasitoids of the fire ant *S. invicta* [39]. Are phorids involved in transmission of these and perhaps other diseases among honey bees in a colony? Are phorids involved in transmission of pathogens between the non-native honey bees and native bees? Alternately, are phorids a dead end for pathogens since as parasitoids they might kill their host before the pathogens can multiply? Answering these questions will require more detailed study. However, just because an infectious agent ultimately proves fatal does not mean it cannot be a vector for other pathogens. This is especially true if the development time of phorid larvae is long. Our results document that phorid-infected foragers spend time in their hive before abandoning it. This period of infection (before abandonment) could extend for a week or more providing time for the pathogens to multiply.

In the case of DWV, the virus has been isolated from the feces and intestines of queen honey bees [40]. If this is true of workers, it provides a potential means to transmit the virus in fluids exchanged by honey bees or by close contact. Vectoring of microsporidian infections during oviposition occurs in some parasitic hymenopteran parasitoids [41], [42]. This mode of transmission has been documented under laboratory conditions for at least three different pathogen-parasitoid-host complexes [42]. Similar to *A. borealis*, *Pseudacteon* phorids have tested positive for microsporidian pathogens of fire ants and have been suggested as a possible vector via oviposition [39]. As yet, it is unclear what proportion of *A. borealis* attacks in the wild result in successful parasitism; however, it is conceivable that unsuccessful attacks could still puncture the abdomen and expose the target bee to any pathogens infecting or carried by the phorid. Considering other honey bee parasites, such as the *Varroa destructor* mite, have been implicated as a vector of DWV, Kashmir bee virus, slow paralysis virus, and Israeli acute paralysis virus, [10]–[12], phorid flies may also act as vectors for DWV or *N. ceranae*. Finally, *N. ceranae* and DWV have been isolated from bumble bees suggesting that exchange of pathogens between honey bees and bumble bees has occurred [43].

*Apocephalus borealis* may also be a threat to native pollinators since it parasitizes a number of bumble bee species and paper wasps (*Vespula* spp) [13], [14]. Wild bumble bees are experiencing substantial declines in North America [44], [45]. So far, attention has focused on emerging pathogens such as *Crithidia bombi and Nosema bombi*. In the laboratory, bumble bees parasitized by *A. borealis* show a dramatic reduction in life span compared to unparasitized bees [13]. The high rate of parasitism in some of our samples of foraging bumble bees and previous high parasitism rates from Canada [13], suggest that parasitism by *A. borealis*, especially in combination with infection by emerging pathogens, could place significant stress on bumble bee populations. If so, phorid parasitism or pathogen transmission to bumble bees might contribute to a cascade of effects in plant and agricultural communities that rely on bumble bees as pollinators. Furthermore, the domestic honey bee is potentially *A. borealis'* ticket to global invasion. Establishment of *A. borealis* on other continents, where its lineage does not occur, where host bees are particularly naïve, and where further host shifts could take place, could have negative implications for worldwide agriculture and for biodiversity of non-North American wasps and bees.

## Methods

### Ethics statement

Samples of San Francisco Bay Area honey bees and bumble bees were obtained with appropriate permissions from beekeepers, landowners and the San Francisco Recreation and Parks Department.

### Sampling procedures

We sampled honey bees from a variety of circumstances. Our main samples consisted of the following: 1) Bees found stranded under lights near the main entrance to Hensill Hall on the San Francisco State University campus (Figure S4A–C). From April 2009 until January 2010, a portion of bees found stranded under lights was sampled at irregular intervals (Range = 2–112 bees per sample). From February to November 2010, stranded bees were sampled at two-day intervals (Range = 2–56 bees per sample) (Figure 3A). All bees were cleared from beneath the lights prior to sundown to ensure that only bees from one night's flight were included in each sample the next morning. Samples consisted of all bees found stranded under the lights. 2) We collected active, foraging bees monthly from our main study hive on the San Francisco State University campus. Samples consisted of 50 incoming foragers collected in individual *Drosophila* vials and samples of 50 or more outgoing foragers collected by placing a standard aerial insect net in front of the hive entrance for 30–60 seconds. We compared the rate of infection in samples of outgoing foragers and incoming foragers. We found no significant difference between these groups (Fishers Exact Test p = 0.32). Therefore, both groups are used to determine long-term trends in rates of infection in active, foraging bees (Figure 3A). This allowed us to compare infection rates of foraging vs. stranded bees. 3) We periodically placed a 1.83 m×1.83 m×1.83 m enclosure (Nicamaka Pop-Up Beach Shade/Tent) over the hive after sunset and removed it before dawn (Fig S4A). We collected all bees captured in the enclosure. Prior to setting up the enclosure, we removed all bees from the area under nearby lights. This allowed us to compare the number of bees stranded under lights during enclosure experiments to the number of bees stranded the day after enclosure experiments. 4) In April 2010, we established an observation hive that allowed us to observe in-hive activities and check for presence of phorids within the hive.

In order to survey prevalence of parasitism in nearby areas, we collected stranded and foraging bees from a variety of locations in the San Francisco Bay Area and from the hives of local beekeepers who agreed to participate in our study. In two of these locations (Table S1 and S2) bees came from areas near feral hives. The feral hive on the San Francisco State University campus has been in place for a number of years and was present before our main study hive appeared on campus. Bees collected near this feral hive were found stranded under a light that is immediately adjacent to the tree containing the colony. The second feral hive was in a tree near the California Academy of Sciences and was discovered during our study. Its history is unknown. We collected stranded bees from beneath the tree that it occupied. In addition, we collected samples of two bumble bee species from the San Francisco Bay Area, *Bombus vosnesenskii* and *B. melanopygus* (Table S1).

### Assessment of parasitism rates

In order to assess parasitism rates, bees from all samples were brought into the laboratory and confined at room temperature (19–20°C) in individual glassine envelopes or *Drosophila* rearing containers from April 2009 to November 2010. We checked confined bees daily for a period of two weeks and recorded the number of phorid larvae that emerged. Additionally, we recorded date of larval emergence for a subset of 636 parasitized bees and duration of the pupal instar for a subset of 94 pupae.

### Laboratory phorid-honey bee infections

In order to observe interactions between phorids and honey bees, adult flies were obtained from a hatching chamber provisioned with a feeder (a 2.54 cm plastic straw filled with cotton saturated in sugar water) and allowed to sit for at least one day in a container provisioned with dishes containing cotton soaked in sugar water and honey solutions. Adult flies were then placed into a clear plastic enclosure approximately 24 cm×12 cm×13.5 cm, and individual honeybees were introduced to them. With each introduction, we recorded whether phorids approached the bee and demonstrated oviposition behavior. After exposure, bees were kept alive in containers provisioned with sugar water and honey solutions.

### Barcode sequencing and phylogenetic comparison

We used DNA barcoding to confirm that the morphologically similar phorids from bumble bees and honey bees were conspecific (Figure S1). High genetic similarity between the two also would support the view that the native *A. borealis* has expanded its host range to include non-native honey bees. We used Qiagen Blood & Tissue DNA extraction kits (Qiagen, Valencia CA) to extract all cellular DNA from collected honey bees, bumble bees, and phorid pupae. We used standard CO1 primers [46] (IDT, Coralville IA)(FWD, 5′ TAAACTTCAGGGTGACCAAAAAATCA…. REV, 5′ GGTCAACAAATCATAAAGATATTG) and the following PCR conditions (1 cycle of 95°C 1 min; 5 cycles of 95°C 1 min, 45°C 1.5 min, 72°C 1.5 min; 35 cycles of 95°C 1 min, 50°C 1.5 min, 72°C 1.5 min; 1 cycle 72°C 5 min) and visualized products on 1% agarose gels. PCR reactions were purified using QiaQuick columns (Qiagen, Valence CA) and sent to Elim Biopharmaceuticals Inc (Hayward, CA) for standard Sanger dideoxy sequencing in both the forward and reverse direction using the CO1 primers. Reads from each orientation were manually contiged using Sequencher (v4.8 Gene Codes Corporation Ann Arbor, MI), and DNA mismatches were visually compared to the DNA chromatogram to correct miscalled bases. Corrected, contigs were aligned using CLUSTALX [47] known phorid barcode sequences and a neighbor-joining tree was generated using 1000 bootstrap replicates.

### Microarray analysis

An Arthropod Pathogen Microarray (APM) [16], [19] including all known honey bee viruses, fungal and bacterial pathogens of honey bees, and mite-specific oligos was augmented with products specific to the phorid 18S rRNA gene. Using phorid larvae, total RNA spiked into unparasitized honey bee total RNA, the PCR assay was capable of detecting one part phorid in 10,000 parts honey bee from 5 ng of cDNA, suggesting that relatively early infections could be detected. In total, 378 samples collected from 2008–2010 were screened, including a 20-hive time-course study sampled approximately biweekly as commercial hives migrated from Mississippi to South Dakota and finally to California (Figure 1). Here, five pooled workers each were screened by PCR and Sanger sequencing of the phorid 18S rRNA gene.

Whole insects were homogenized in 1 mL of 1∶1 Trizol∶PBS with a 5 mm steel ball in a TissueLyzer II at 30 Hz for 4 min. Total nucleic acid was extracted by the addition of 100 µL chloroform and centrifugation, followed by isopropanol precipitation. For each sample, one quarter of the total nucleic acid (1–5 µg) was randomly primed with Superscript II (Invitrogen) with primer RdA (5′GTTTCCCACTGGAGGATANNNNNNNNN). Second-strand synthesis was performed twice with the same primer and Sequenase DNA polymerase (USB). One quarter of this reaction was amplified with Taq polymerase and a single adapter primer RdB (5′GTTTCCCACTGGAGGATA). This randomly amplified material was used for screening for Phorid rRNA with primer pair Phorid-rRNA-1F (GTACACCTATACATTGGGTTCGTACATTAC) and -1R (GAGRGCCATAAAAGTAGCTACACC) in a Taq polymerase PCR with an annealing temperature of 57°C.

For pathogen detection by microarray, the randomly amplified material was further amplified and labeled with a dye-linked primer RdC (5′Cy3-GTTTCCCACTGGAGGATA), column purified and hybridized to a 70-mer DNA microarray in 3× SSC, 50 mM HEPES and 0.5% SDS at 65°C overnight. Microarrays were scanned on an Axon 4000A scanner and analyzed visually or with the Cluster analysis package [48]. All microarray spots that indicated the presence of pathogens were further confirmed by PCR and Sanger sequencing with primers *Nosema ceranae* F-4186 (5′-CGGATAAAAGAGTCCGTTACC) and R-4435 (5′-TGAGCAGGGTTCTAGGGAT) [49] and DWV-F-1165 (5′-CTTACTCTGCCGTCGCCCA) -R-1338 (5′-CCGTTAGGAACTCATTATCGCG) [50].

### Data availability and compliance with standards

The *A. borealis* mitochondrial barcode sequence (ID# JF798506) and18S rRNA gene sequence (ID# JF808447) have been deposited in Genbank. APM design and results have been submitted to GEO (design accession GPL11490 and array data accession GSE28235) and are MIAME compliant.

## Supporting Information

Figure S1 **CLUSTALX alignment of 450 bp of cytochrome oxidase I DNA barcodes obtained from infected honey bees (samples 19–24,26–31,34,35) and bumble bees (samples 33,36).** Bidirectional Sanger sequence indicates that only two positions varied (88, 288) in a single sample each. All samples had less than 0.22% divergence (i.e. 1 bp).
(PDF)

Figure S2 ***A. borealis*** **18S rRNA and mitochondrial cytochrome oxidase I (COI) DNA sequence used for barcoding and APM.**
(PDF)

Figure S3 **Timing of life history events in parasitism of honey bees by** ***A. borealis*** **.** (A) Length of time after sample collection until phorid larvae emerged from their honey bee hosts (Mean = 7.14 days, SD = 1.68, n = 636). (B) Number of phorid larvae per infected bee for samples from various locations (Mean = 4.8, SD = 2.45, n = 961). (C) Length of pupal period (Mean = 27.9 days, SD = 1.9, n = 94).
(PDF)

Figure S4 **San Francisco State University Hensill Hall study site.** (A) Primary study hive, blue arrow indicates direction that honey bees fly to reach the nearby light. (B) Landing above the hive where stranded bees were collected and the light (C) immediately above the landing showing honey bees attracted to it from the previous night. (D) A typical enclosure setup.
(PDF)

Figure S5 **The number of parasitized bees (red) compared to all bees (black) collected at the San Francisco State University Hensill Hall collection site.** Notably, numerous bees were collected from the lights and landing in months even when parasitism rate was low. Our direct rearing method may have underestimated the rate of parasitism during spring 2010 since the Arthropod Pathogen Array (APM) indicated a higher rate of parasitism during April and early May than we observed in our rearings. The APM also detected a high level of infection with *Nosema ceranae* and deformed wing virus during that period.
(PDF)

Table S1 **Honey bee and bumble bee collection sites in the San Francisco Bay Area.** Locations of hives which did not yield parasitism in the San Francisco Bay Area are shaded light grey. Locations where stranded and foraging honey bees and bumble bees were collected are shaded dark grey.
(PDF)

Table S2 **Arthropod Pathogen Microarray results.** Location codes are main study hive (HHH), stranded on landing near main hive (HHL), main hive enclosure (HHC), observation hive (OH), near feral hive on San Francisco State University campus (GYMA), feral hive near California Academy of Sciences (CAS), X's indicate whether infected by phorids, *Nosema ceranae*, or deformed wing virus.
(PDF)

Table S3 **Rate of parasitism for** ***Bombus vosnesenskii*** **sampled from San Francisco, California locations from May to November 2010.**
(PDF)
