# Supplementary material for: A New Threat to Honey Bees, the Parasitic Phorid Fly Apocephalus borealis
Source: PLoS One. 2012 Jan 3;7(1):e29639. doi: 10.1371/journal.pone.0029639 (PMC3250467; doi:10.1371/journal.pone.0029639)

Figure S1: CLUSTALX alignment of 450bp of cytochrome oxidase I DNA barcodes obtained from infected honey bees (samples 19-24,26-31,34,35) and bumble bees (samples 33,36). Bidirectional Sanger sequence indicates that only two positions varied (88, 288) in a single sample each. All samples had less than 0.22% divergence (i.e. 1bp).

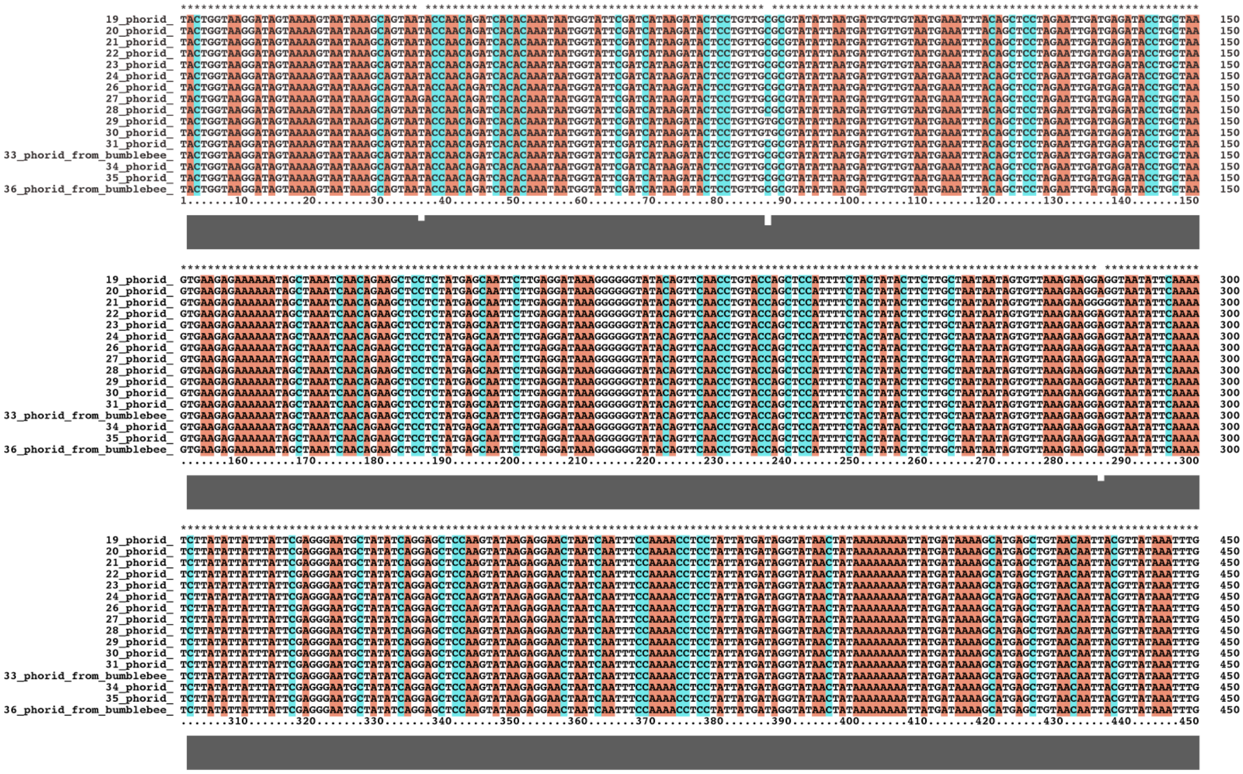

Supplement: Figure S1 — CLUSTALX alignment of 450 bp of cytochrome oxidase I DNA barcodes obtained from infected honey bees (samples 19–24,26–31,34,35) and bumble bees (samples 33,36). Bidirectional Sanger sequence indicates that only two positions varied (88, 288) in a single sample each. All samples had less than 0.22% divergence (i.e. 1 bp). (PDF) [file pone.0029639.s001.pdf]
