# Supplementary material for: A New Threat to Honey Bees, the Parasitic Phorid Fly Apocephalus borealis
Source: PLoS One. 2012 Jan 3;7(1):e29639. doi: 10.1371/journal.pone.0029639 (PMC3250467; doi:10.1371/journal.pone.0029639)
Supplement: Figure S2 — A. borealis 18S rRNA and mitochondrial cytochrome oxidase I (COI) DNA sequence used for barcoding and APM. (PDF) [file pone.0029639.s002.pdf]

Figure S2. ***Apocephalus borealis*** **18S rRNA and mitochondrial cytochrome oxidase I (COI) DNA sequence used for barcoding and APM.**

>Phorid_rRNA_Consensus

GGAATGAGWACACTTTAAATCCTTTAACAAGGACCTATTGGAGGGCAAGTCTGGTGCCA

GCAGCCGCGGTAATTCCAGCTCCAATAGCGTATATTAAAGTTGTTGCGGTTAAAACGTT

CGTAGTTGAACTTGTGCTCCATACGGGTAGTACACCTATACATTGGGTTCGTACATTAC

TCTATGGTATGTGAGCGTATTACCGGTGGAGTTCTTATATGTAATAGTACTTGTATTAT

TGCATATTCCTCCTATTCAAACCTACTTCMGTGCTCTTCACCGAGTGTTGTTGTGGGCC

GGTACAATTACTTTGAACAAATTAGAGTGCTTAAAGCAGGCTTCAAATGCCTGAATATT

TGTGCATGGAATAATGGAATAAGACCTCTGTTCTACTTTCATTGGTTTATAGATCAAGA

GGTAATGATTAATAGAAGCAGTTTGGGGGCATTAGTATTACGACGCGAGAGGTGAAATT

CTTGGACCGTCGTAAGACTAACTTAAGCGAAAGCATTTGCCAAAGATGTTTTCATTAAT

CAAGAACGAAAGTTAGAGGTTCGAAGGCGATCAGATACCGCCCTAGTTCTAACCATAAA

CGATGCCAGCTAGCAATTGGGTGTAGCTACTTTTATGGCYCTCTCAGTCGCTTCCCGGG

AAACCAAAGCTTTTGGGCTCCGGGGGAAGTATGGTTGCAAAGCTGAAACTTAAAGGAAT

TGACGGAAGGGCACCACCAGGAGTGGAGCCTGCGGCTTAATTTGACTCAACACGGGAAA

ACTTACCAGGTCCGAACATAAATGAGTAWGACAGATTGATAGCTCTTTCTCGAATCTAT

GGGTGGTGGTGCATGGCCGTTCTTAGTTCGTGGAGTGATTTGTCTGGTTAATTCCGATA

AYGAACGAGACTCAAATATATTAAATAGATGCTTTCAGGATTATAGCGTTGAAACTTAT

ATATCGGTCRGCATGAATGCAGTAACATGTCCTAGTGTTTGATTTGATTATATARGTGG

AGTTGTACCTGTTGGTTTGTCCCATTATAAGGACACTAGCTTCTTAAATGGACAAATTG

CGTCTAGCAATAATGAGATTGAGCAATAACAGGTCTGTGATGCCCTTAGATGTCCTGGG

CTGCACGCGCGCTACAATGAAAGTATCAACGTGTATTTCCTAGACCGAGAGGTCCGGGT

AAACCGCTGAACCACTTTCTTGCTTGGGATTGTGAACTGAAACTGTTCACATGAACCTG

GAATTCCCAGTAAGTGTGAGTCATTAACTCGCATTGATTACGTCCCTGC

>Phorid_COI

TTATCCAGATGCTTATACCTCCTGAAATATTATCTCCTCTATTGGTTCATATGTTTCAT

TTTTAAGAATTATCTTTTTCTTATATATTATCTGAGAAAGAATAACTTCACAACGTCTA

CTTATTACCCCTTCACAAATAAGTTCATCTATTGAATGATACCAAAATACTCCTCCCTC

TGAACATAGTTATTCTGAACTACCTTTATTAACAAATTAAATCTAACGTGGCAGATTAG

TGCAATGGATTTAAGCTCCAAATATAAAGTATTTAACTTTCATTAGAACACAAATGTCA

ACTTGAGCTAATTTAAATTTACAAGATAGTGCATCACCATTAATAGAACAATTAACCTT

CTTTCATGATCATACACTATTAATTTTAGTATTAATTACTGTTTTAGTTTCTTATATAA

TATCAATTTTATTTTTTAACAGTTTCTCAAATCGATTCTTGTTACACGGACAAATAATT

GAAATCATTTGAACTATTCTACCAGCTATAACTTTGTTATTCATTGCTTTTCCCTCTCT

TCGTCTATTATATTTAATTGACGAAATTAATGAACCAATAATTACCTTAAAATCAATTG

GCCAC
