# Supplementary material for: A New Threat to Honey Bees, the Parasitic Phorid Fly Apocephalus borealis
Source: PLoS One. 2012 Jan 3;7(1):e29639. doi: 10.1371/journal.pone.0029639 (PMC3250467; doi:10.1371/journal.pone.0029639)

Figure S3. Timing of life history events in parasitism of honey bees by *A. borealis*. (A) Length of time after sample collection until phorid larvae emerged from their honey bee hosts (Mean = 7.14 days, SD = 1.68, n = 636). (B) Number of phorid larvae per infected bee for samples from various locations (Mean = 4.8, SD = 2.45, n = 961). (C) Length of pupal period (Mean = 27.9 days, SD = 1.9, n = 94).

A

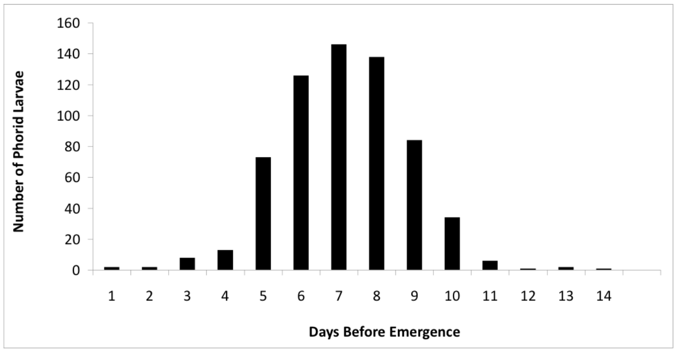

B

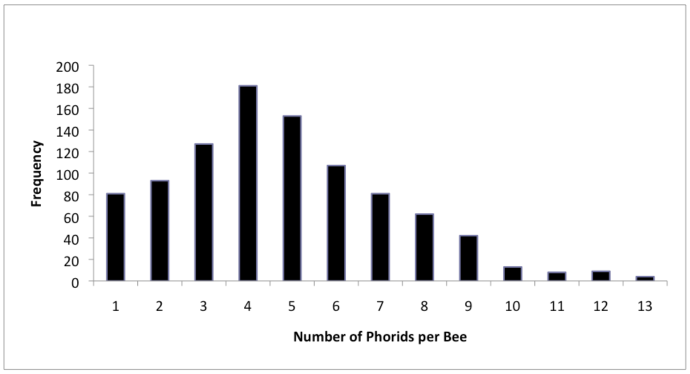

C

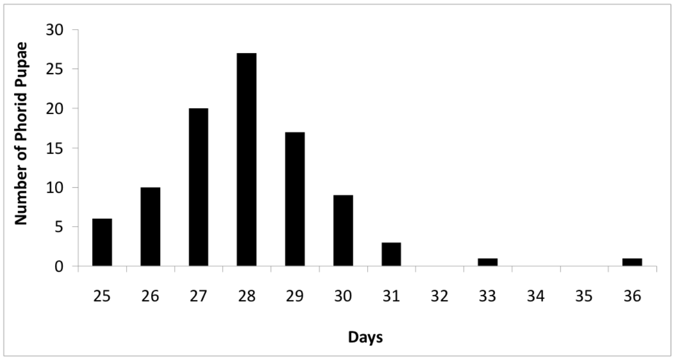

Supplement: Figure S3 — Timing of life history events in parasitism of honey bees by A. borealis . (A) Length of time after sample collection until phorid larvae emerged from their honey bee hosts (Mean = 7.14 days, SD = 1.68, n = 636). (B) Number of phorid larvae per infected bee for samples from various locations (Mean = 4.8, SD = 2.45, n = 961). (C) Length of pupal period (Mean = 27.9 days, SD = 1.9, n = 94). (PDF) [file pone.0029639.s003.pdf]
