# Supplementary material for: A New Threat to Honey Bees, the Parasitic Phorid Fly Apocephalus borealis
Source: PLoS One. 2012 Jan 3;7(1):e29639. doi: 10.1371/journal.pone.0029639 (PMC3250467; doi:10.1371/journal.pone.0029639)

Figure S4. San Francisco State University Hensill Hall study site.
(A) Primary study hive, blue arrow indicates direction that honey bees fly towards the nearby light. (B) Landing above the hive where stranded bees were collected and the light. (C) immediately above the landing showing honey bees attracted to it from the previous night. (D) A typical enclosure setup.

A.

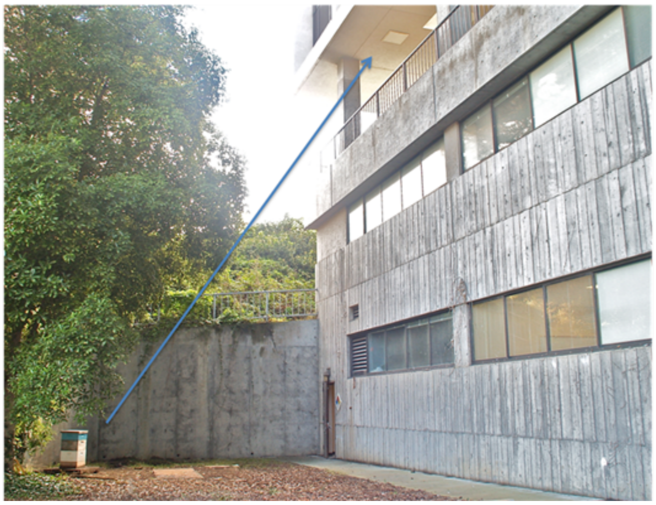

B.

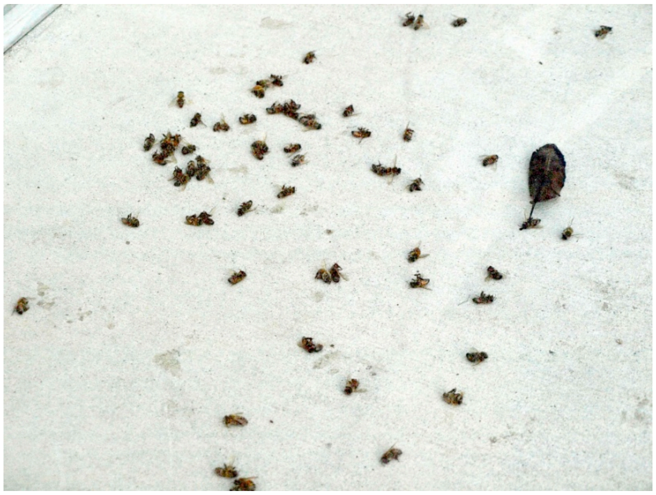

C.

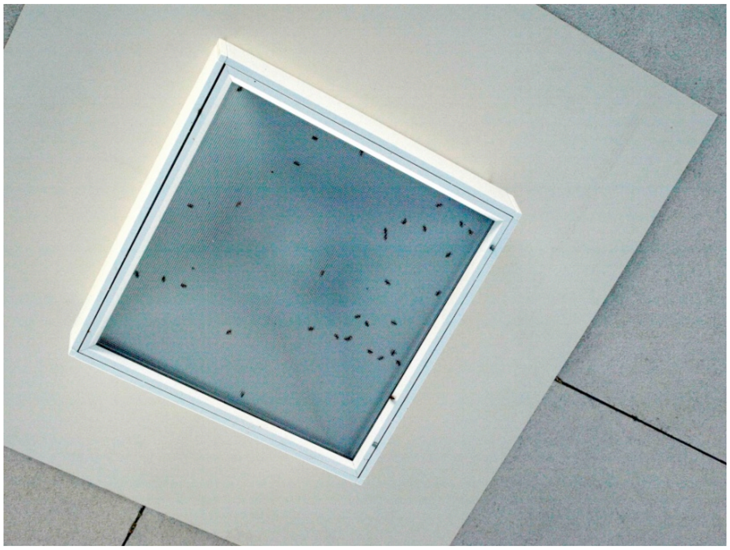

D.

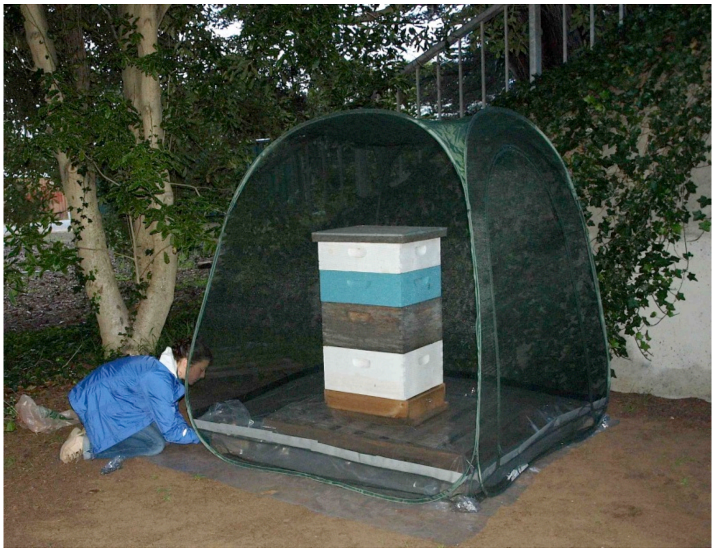

Supplement: Figure S4 — San Francisco State University Hensill Hall study site. (A) Primary study hive, blue arrow indicates direction that honey bees fly to reach the nearby light. (B) Landing above the hive where stranded bees were collected and the light (C) immediately above the landing showing honey bees attracted to it from the previous night. (D) A typical enclosure setup. (PDF) [file pone.0029639.s004.pdf]
