# Supplementary material for: A New Threat to Honey Bees, the Parasitic Phorid Fly Apocephalus borealis
Source: PLoS One. 2012 Jan 3;7(1):e29639. doi: 10.1371/journal.pone.0029639 (PMC3250467; doi:10.1371/journal.pone.0029639)

Figure S5. The number of parasitized bees (red) compared to all bees (black) collected at the San Francisco State University **Hensill Hall collection site.** Notably, numerous bees were collected from the lights and landing in months even when parasitism rate was low. Our direct rearing method may have underestimated the rate of parasitism during spring 2010 since the Arthropod Pathogen Array (APM) indicated a higher rate of parasitism during April and early May than we observed in our rearings. The APM also detected a high level of infection with *Nosema ceranae* and deformed wing virus during that period.

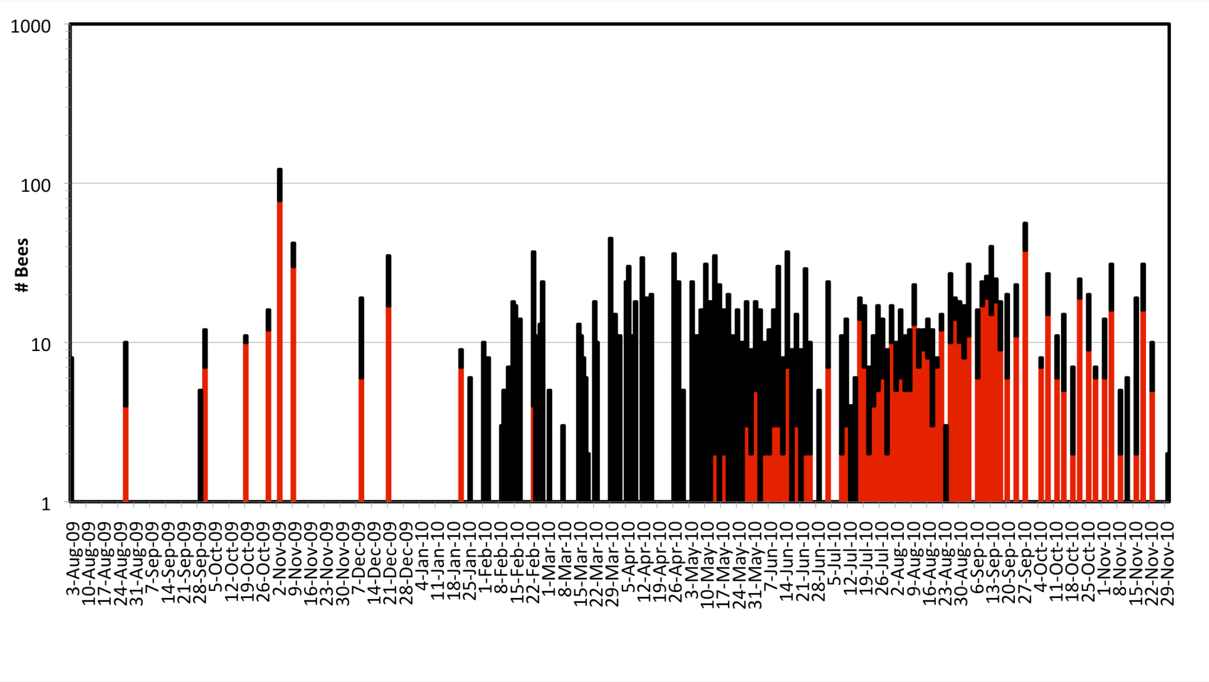

Supplement: Figure S5 — The number of parasitized bees (red) compared to all bees (black) collected at the San Francisco State University Hensill Hall collection site. Notably, numerous bees were collected from the lights and landing in months even when parasitism rate was low. Our direct rearing method may have underestimated the rate of parasitism during spring 2010 since the Arthropod Pathogen Array (APM) indicated a higher rate of parasitism during April and early May than we observed in our rearings. The APM also detected a high level of infection with Nosema ceranae and deformed wing virus during that period. (PDF) [file pone.0029639.s005.pdf]
