# Supplementary material for: A New Threat to Honey Bees, the Parasitic Phorid Fly Apocephalus borealis
Source: PLoS One. 2012 Jan 3;7(1):e29639. doi: 10.1371/journal.pone.0029639 (PMC3250467; doi:10.1371/journal.pone.0029639)
Supplement: Table S1 — Honey bee and bumble bee collection sites in the San Francisco Bay Area. Locations of hives which did not yield parasitism in the San Francisco Bay Area are shaded light grey. Locations where stranded and foraging honey bees and bumble bees were collected are shaded dark grey. (PDF) [file pone.0029639.s006.pdf]

Table S1. Honey bee and bumble bee collection sites in the San Francisco Bay Area. Locations of hives which did not yield parasitism in the San Francisco Bay Area are shaded light grey. Locations where stranded and foraging honey bees and bumble bees were collected are shaded dark grey.

Location	County	Species	# Bees Sampled	Phorids Detected	Notes
SF State, Hensill Hall, 94132	San Francisco	*A. mellifera*	2174	+	Sampled from under lights, adjacent to main study hive
			435	+	Sampled from enclosures on main study hive
			795	+	Sampled from main study hive
SF State 5th Floor Stairwell Breezeway Hensill Hall 94132	San Francisco	*A. mellifera*	18	+	Sampled from under lights
SF State, Hensill Hall, Greenhouse 94132	San Francisco	*A. mellifera*	574	+	Sampled from under lights at greenhouse
SF State, Hensill Hall, Observation Hive 94132	San Francisco	*A.mellifera*	30	+	Sampled bees from within hive entrance
SF State, Feral Hive, Hensill Hall 94132	San Francisco	*A.mellifera*	20	+	Sampled from under lights near hive
Golden Gate Park (GGP) Apiary 94122	San Francisco	*A. mellifera*	16	+	Sampled from area around hives
Heron's Head Park 94124	San Francisco	*A. mellifera*	213	+	Sampled foragers
Van Buren St. 94131	San Francisco	*A. mellifera*	323	+	Sampled foragers and from area around hives
17th St. and Dolores 94110	San Francisco	*A. mellifera*	36	+	Sampled from area around hives
Laguna and Oak 94102	San Francisco	*A. mellifera*	81	+	Enclosure and sampled foragers
7th St. and Lawton 94122	San Francisco	*A. mellifera*	88	+	Sampled foragers -
12th St. and Lake 94118	San Francisco	*A. mellifera*	247	+	Sampled foragers and light trap
Hickory and Octavia 94102	San Francisco	*A. mellifera*	36	+	Sampled foragers
Mizpah St. and Sussex 94131	San Francisco	*A. mellifera*	5	+	Collected in a light trap
SF Insect Zoo 94132	San Francisco	*A. mellifera*	5	+	Sampled from area around hive

El Sobrante, CA 94803	Contra Costa	*A. mellifera*	288	+	Sampled foragers and from area around hives
Walnut Creek, CA 94595	Contra Costa	*A. mellifera*	326	+	Sampled foragers and from area around hives
Mill Valley, CA 94941	Marin	*A. mellifera*	414	+	Sampled foragers and from area around hives
Orinda, CA 94563	Contra Costa	*A. mellifera*	340	+	Sampled foragers and from area around hives
Rio Vista, CA 94571	Solano	*A. mellifera*	248	+	Sampled foragers
Pleasant Hill, CA 94523	Contra Costa	*A. mellifera*	196	+	Sampled foragers
Oakland, CA 94601	Alameda	*A. mellifera*	254	+	Sampled foragers and from area around hives
Oakland, CA 94602	Alameda	*A. mellifera*	295	+	Sampled foragers and from area around hives
Concord, CA 94519	Contra Costa	*A. mellifera*	185	+	Sampled foragers and from area around hives
Martinez, CA 94553	Contra Costa	*A. mellifera*	344	+	Sampled foragers and from area around hives
26th St. and Douglass 94114	San Francisco	*A. mellifera*	61	-	Sampled foragers and from area around hives
Bryant and 26th St. 94110	San Francisco	*A. mellifera*	73	-	Sampled foragers and from area around hives
Islais Creek Park 94124	San Francisco	*A. mellifera*	71	-	Sampled foragers and from area around hives
Alamo, CA 94507	Contra Costa	*A. mellifera*	78	-	Sampled foragers and from area around hives
San Ramon, CA 94583	Contra Costa	*A. mellifera*	56	-	Samples from area around hives
San Jose, CA 95127	Santa Clara	*A. mellifera*	480	-	Sampled foragers
Antioch, CA 94509	Contra Costa	*A. mellifera*	41	-	Sampled foragers and from area around hives

McLaren Park 94134	San Francisco	*A. mellifera*	28	+	Sampled foragers
Clarendon St. 94131	San Francisco	*A. mellifera*	19	+	Discovered stranded on ground
JFK Drive (GGP) 94122	San Francisco	*A. mellifera*	1	+	Discovered stranded on ground
Sutro Tower 94131	San Francisco	*A. mellifera*	2	+	Discovered stranded on ground
SF State, Hensill Hall Greenhouse 94132	San Francisco	*Bombus vosnesenskii*	4	+	Sampled foragers
19th Ave. and Vicente 94116	San Francisco	*Bombus vosnesenskii*	45	+	Sampled foragers
Lake Merced 94132	San Francisco	*Bombus vosnesenskii*	47	+	Sampled foragers
14th Ave. and Balboa 94118	San Francisco	*Bombus vosnesenskii*	10	+	Sampled foragers
McLaren Park 94134	San Francisco	*Bombus vosnesenskii*	19	+	Sampled foragers
Mizpah St. and Sussex 94131	San Francisco	*Bombus vosnesenskii*	1	+	Sampled foragers
Quintara St. and Cragmont 94116	San Francisco	*A. mellifera*	1	-	Discovered stranded on ground
Glen Canyon Park 94131	San Francisco	*A. mellifera*	5	-	Discovered stranded on ground
12th St. and Fulton 94118	San Francisco	*A. mellifera*	1	-	Discovered stranded on ground
Glen Canyon Park 94131	San Francisco	*Bombus vosnesenskii*	2	-	Sampled foragers
8th Ave. and Lake 94118	San Francisco	*Bombus vosnesenskii*	18	-	Sampled foragers
California Academy of Sciences (GGP) 94118	San Francisco	*Bombus vosnesenskii*	29	-	Sampled foragers
Carl St. and Arguello 94122	San Francisco	*Bombus vosnesenskii*	1	-	Sampled foragers
17th Ave. and Stanyan 94117	San Francisco	*Bombus vosnesenskii*	1	-	Sampled foragers
8th Ave. and Lake 94118	San Francisco	*Bombus melanopygus*	8	+	Sampled foragers
California Academy of Sciences (GGP) 94118	San Francisco	*Bombus melanopygus*	3	-	Sampled foragers

12th Ave. and Fulton 94118	San Francisco	*Bombus melanopygus*	1	-	Sampled foragers
Belgrave St. and Stanyan 94112	San Francisco	*Bombus melanopygus*	1	-	Sampled foragers
McLaren Park 94134	San Francisco	*Bombus melanopygus*	1	-	Sampled foragers
19th Ave. and Vicente 94116	San Francisco	*Bombus melanopygus*	4	-	Sampled foragers
