# Supplementary material for: A New Threat to Honey Bees, the Parasitic Phorid Fly Apocephalus borealis
Source: PLoS One. 2012 Jan 3;7(1):e29639. doi: 10.1371/journal.pone.0029639 (PMC3250467; doi:10.1371/journal.pone.0029639)
Supplement: Table S2 — Arthropod Pathogen Microarray results. Location codes are main study hive (HHH), stranded on landing near main hive (HHL), main hive enclosure (HHC), observation hive (OH), near feral hive on San Francisco State University campus (GYMA), feral hive near California Academy of Sciences (CAS), X's indicate whether infected by phorids, Nosema ceranae, or deformed wing virus. (PDF) [file pone.0029639.s007.pdf]

Table S2. Arthropod Pathogen Microarray results. Location codes are main study hive (HHH), stranded on landing near main hive (HHL), main hive enclosure (HHC), observation hive (OH), near feral hive on San Francisco State University campus (GYMA), feral hive near California Academy of Sciences (CAS), X's indicate whether infected by phorids, *Nosema ceranae*, or deformed wing virus.

Description	*Apocephalus borealis*	*Nosema ceranae*	Deformed Wing Virus	Location	Date
Honey bee	X	X	X	OH	Apr 23, 2010
Honey bee				OH	Apr 23, 2010
Honey bee				OH	Apr 23, 2010
Honey bee			X	OH	Apr 23, 2010
Honey bee				OH	Apr 23, 2010
Honey bee		X		HHL	Apr 26, 2010
Honey bee		X		HHL	Apr 26, 2010
Honey bee		X		HHL	Apr 26, 2010
Honey bee	X	X	X	HHL	May 3, 2010
Honey bee	X	X	X	HHH	May 26, 2010
Honey bee		X		HHH	May 26, 2010
Honey bee		X		HHH	May 26, 2010
Honey bee	X	X	X	HHC	May 27, 2010
Honey bee	X	X	X	HHC	May 27, 2010
Honey bee		X		HHC	May 27, 2010
Honey bee		X		HHC	May 27, 2010
Honey bee		X		HHC	May 27, 2010
Honey bee				HHL	May 28, 2010
Honey bee				HHL	May 28, 2010
Honey bee		X	X	GYMA	June 2, 2010
Honey bee		X		GYMA	June 2, 2010
Honey bee		X	X	CAS	June 2, 2010
Honey bee			X	CAS	June 2, 2010
Honey bee		X	X	CAS	June 2, 2010
Honey bee		X	X	CAS	June 2, 2010
Honey bee		X	X	CAS	June 2, 2010
Honey bee	X	X		HHH	June 7, 2010
Honey bee	X	X	X	HHH	June 7, 2010

Honey bee	X	X	X	HHL	June 8, 2010
Honey bee				HHL	June 8, 2010
Honey bee				HHL	June 8, 2010
Honey bee				HHL	June 8, 2010
Honey bee		X		HHL	June 18, 2010
Honey bee		X	X	HHL	June 2010
Honey bee	X	X	X	HHH	June 2010
Honey bee	X	X		HHL	June 2010
Phorid Adult	X			Raised from Bee	April 12, 2010
Phorid Adult	X			Raised from Bee	April 12, 2010
Phorid Larva	X	X		Emerged from Bee	May 3, 2010
Phorid Larva	X	X	X	Emerged from Bee	May 3, 2010
Phorid Larva	X		X	Emerged from Bee	May 3, 2010
Phorid Adult	X	X		Raised from Bee	May 11, 2010
Phorid Larva	X	X		Emerged from Bee	May 17, 2010
Phorid Larva	X	X	X	Emerged from Bee	May 17, 2010
Phorid Larva	X	X	X	Emerged from Bee	May 17, 2010
Phorid Larva	X	X	X	Emerged from Bee	May 17, 2010
Phorid Larva	X	X	X	Emerged from Bee	May 17, 2010
Phorid Adult	X	X	X	Raised from Bee	May 22, 2010
Phorid Adult	X	X		Raised from Bee	May 22, 2010
Phorid Adult	X			Raised from Bee	May 2010
Phorid Adult	X		X	Raised from Bee	May 2010
Phorid Adult	X	X		Raised from Bee	May 2010
