# Supplementary material for: A New Threat to Honey Bees, the Parasitic Phorid Fly Apocephalus borealis
Source: PLoS One. 2012 Jan 3;7(1):e29639. doi: 10.1371/journal.pone.0029639 (PMC3250467; doi:10.1371/journal.pone.0029639)
Supplement: Table S3 — Rate of parasitism for Bombus vosnesenskii sampled from San Francisco, California locations from May to November 2010. (PDF) [file pone.0029639.s008.pdf]

**Table S3. Rate of parasitism for *Bombus vosnesenskii* sampled from San Francisco, California locations from May to November 2010.**

| Date of Collection | Rate of Parasitism | Species | # of Bees Sampled |
|---|---|---|---|
| 24-May-10 | 0.21 | *Bombus vosnesenskii* | 19 |
| 3-Jun-10 | 0.23 | *Bombus vosnesenskii* | 13 |
| 15-Jun-10 | 0.18 | *Bombus vosnesenskii* | 11 |
| 24-Jun-10 | 0.14 | *Bombus vosnesenskii* | 7 |
| 28-Jun-10 | 0.20 | *Bombus vosnesenskii* | 10 |
| 1-Sep-10 | 0.80 | *Bombus vosnesenskii* | 10 |
| 28-Nov-10 | 1.00 | *Bombus vosnesenskii* | 1 |
